# Comparison of readmissions among hospitalized nonvalvular atrial fibrillation patients treated with oral anticoagulants in the United States

**DOI:** 10.1080/21556660.2020.1750418

**Published:** 2020-04-24

**Authors:** Steven Deitelzweig, Christine L. Baker, Amol D. Dhamane, Jack Mardekian, Oluwaseyi Dina, Lisa Rosenblatt, Cristina Russ, Tayla Poretta, Melissa Lingohr-Smith, Jay Lin

**Affiliations:** a Department of Hospital Medicine, Ochsner Clinic Foundation, New Orleans, LA, USA; b Ochsner Clinical School, The University of Queensland School of Medicine, New Orleans, LA, USA; c Pfizer, Inc, New York, NY, USA; d Bristol-Myers Squibb, Lawrenceville, NJ, USA; e Novosys Health, Green Brook, NJ, USA

**Keywords:** Atrial fibrillation, oral anticoagulants, hospital readmissions, major bleeding

## Abstract

**Objectives:**

To compare the risks of 1-month all-cause, major bleeding (MB)-related and stroke-related readmissions and the associated hospital resource use and costs among patients previously hospitalized for nonvalvular atrial fibrillation (NVAF) and treated with warfarin, rivaroxaban, and dabigatran vs apixaban.

**Methods:**

Adult patients hospitalized with NVAF (any discharge diagnosis position) who received apixaban, warfarin, rivaroxaban, or dabigatran during hospitalization were identified from the Premier database (1 January 2013–30 June 2017) and grouped into respective cohorts. Propensity score matching was used to generate cohorts with similar characteristics. In regression analyses the risk of readmissions that occurred within 1 month of discharge were evaluated and the associated length of stay (LOS) and costs compared.

**Results:**

NVAF patients treated with warfarin vs apixaban had significantly greater risk of all-cause (odds ratio [OR] = 1.05; confidence interval [CI] = 1.02–1.08; *p* < .001), MB-related (OR: 1.28; CI: 1.16–1.42; *p* < .001), and stroke-related (OR: 1.33; CI: 1.11–1.58; *p* = .002) readmissions; for all readmission categories, average LOS was significantly longer and costs significantly higher for warfarin treated patients. NVAF patients treated with rivaroxaban versus apixaban had significantly greater risk of all-cause (OR: 1.06; CI: 1.02–1.09; *p* = .001) and MB-related (OR = 1.62; CI = 1.44–1.83; *p* < .001) readmissions, but not stroke-related readmission; for MB-related readmissions average LOS and costs were higher for rivaroxaban treated patients. Significant differences in risks of all-cause, MB-related, and stroke-related readmissions were not observed between the apixaban and dabigatran cohorts.

**Conclusion:**

In this retrospective real-world analysis of NVAF patients, apixaban treatment was associated with better clinical outcomes than warfarin or rivaroxaban and lower hospital resource burden.

## Introduction

Nonvalvular atrial fibrillation (NVAF) is a cardiac arrhythmia associated with an increased risk for ischemic stroke and systemic embolism and its prevalence is increasing in the US^1^^,^^2^. Largely related to hospitalizations, the economic burden of NVAF has been predicted to approach $30 billion annually by 2050 in the US^3^. In the last decade, several direct-acting oral anticoagulants (DOACs) have been approved by the Food and Drug Administration (FDA) and emerged in the US market for the purpose of reducing stroke risk among patients with NVAF. These DOACs include dabigatran, rivaroxaban, apixaban, and edoxaban, all of which are associated with fewer usage limitations than warfarin, the oral anticoagulant (OAC) that has been prescribed for the past 50 years for stroke risk reduction in NVAF patients^4^^,^^5^.

In large randomized clinical trials of patients with NVAF, DOACs have exhibited noninferior or superior efficacy for the prevention of stroke compared with warfarin^6–8^. Furthermore, depending on the DOAC investigated in the clinical trial, they were associated with lower or similar rates of bleeding relative to warfarin^6–8^. Other studies conducted in the real-world setting have more recently provided evidence that apixaban is associated with lower risks for stroke and major bleeding (MB) than warfarin, as well as lower risks for MB than rivaroxaban and dabigatran; although there is some variation in the results regarding the DOAC comparisons^9–19^. A network meta-analysis published in 2018 of 11 studies conducted in the real-world setting has summarized that, of the OACs (warfarin, dabigatran, rivaroxaban, and apixaban), apixaban is associated with the lowest risk for MB (apixaban vs warfarin hazard ratio [HR] = 0.58; 95% confidence interval [CI] = 0.48–0.69; apixaban vs dabigatran HR = 0.73; 95% CI = 0.61–0.87; apixaban vs rivaroxaban HR = 0.55; 95% CI = 0.46–0.66)^15^. Directionally consistent results in regard to bleeding outcomes have been reported in other meta-analyses of real-world data^16–19^.

In addition to examining the comparative efficacy and safety of OACs in the real-world setting, it is important to determine whether their use may differentially impact hospital resource use, specifically rehospitalization, as well as economic outcomes of NVAF patients. We previously conducted an early investigation of the rates of all-cause and bleeding-related hospital readmissions among hospitalized NVAF patients treated with dabigatran, rivaroxaban, and apixaban (1 January 2012–31 March 2014)^20^. In this previous study of 74,730 patients, 15% were readmitted to the hospital for any cause within 1 month of discharge^20^. After controlling for differences in patient characteristics, the risk of bleeding-related hospital readmission was 40% greater (*p* < .01) for patients who were treated with rivaroxaban and 20% numerically trending greater (*p* = .16) for patients who were treated with dabigatran compared to those treated with apixaban^20^. Herein, we have extended this early investigation to include, in addition to hospitalized NVAF patients treated with the different DOACs, also patients treated with warfarin between January 2013 and September 2017. The objectives of this current study were to compare the risks of 1-month all-cause, MB-related, and stroke-related readmissions and the associated hospital resource use and costs among patients previously hospitalized for NVAF and treated with warfarin, rivaroxaban, and dabigatran vs apixaban.

## Methods

### Study population

Patients (≥18 years of age) hospitalized for NVAF, based on any discharge *International Classification of Diseases, Ninth Revision, Clinical Modification* (ICD-9/10) code indicating NVAF, were identified from the Premier Hospital database between 1 January 2013 and 30 June 2017. The overall study period was from 1 January 2012 to 30 September 2017, to allow for a follow-up period for observation of readmissions and a 1-year baseline observation period. The Premier Hospital database provides hospital billing information on a patient’s hospital stay as well as information on ICD-9/10 codes and current procedural terminology (CPT) codes. Specifically, the database contains date-stamped records of all billed items, including medications, laboratory, diagnostic, and therapeutic services, and primary and secondary diagnoses for each patient’s hospitalization. Identifier-linked files provide demographic and payer information. The Premier Hospital database is a nationally representative inpatient hospitalization records database capturing more than 45 million hospital discharges, ∼ 20% of all hospital admissions in the US, from greater than 700 acute care hospitals. The patient data from this source are de-identified and, thus, in compliance with the Health Insurance Portability and Accountability Act.

The first of such NVAF hospitalizations was defined as the index hospitalization. The discharge date of the index hospitalization was defined as the index date for the evaluation of hospital readmissions. Patients who received apixaban, warfarin, rivaroxaban, or dabigatran at any time during the hospitalization (from admission to discharge) were identified and grouped into study cohorts based on the OAC initiated. Patients with usage of more than one type of OAC drug during index hospitalization were excluded so that patients could be exclusively assigned into each of the OAC patient cohorts. Patients were also excluded from the study cohorts if, during the 12-month baseline period or index hospitalization, they had a medical claim indicative of valvular heart disease, kidney disease, venous thromboembolism, or transient AF; if they had hip or knee surgery within a 6-week period prior to the index date; or if they had a claim indicating pregnancy at any time during the study period. In addition, patients who received edoxaban during the baseline period or index hospitalization were excluded due to its later entry into the market and thus small sample size. Patients who did or did not receive antiplatelet medications were allowed in the study population.

### Demographics, patient clinical characteristics, and hospital characteristics

Demographics, patient clinical characteristics, and hospital characteristics were measured during the index hospitalization. Additionally, the proportions of patients with prior bleeding and stroke were measured during the 12-month baseline period.

### Hospital readmissions

The proportions of patients treated with apixaban, warfarin, rivaroxaban, or dabigatran, along with the all-cause, MB-related, and stroke-related readmission that occurred within 1 month of discharge of initial hospitalization were determined and compared between each of the OAC cohorts and the apixaban cohort in separate comparisons. Presentations to the emergency department not followed by an inpatient admission were not considered as inpatient readmissions in the Premier Hospital database or in this study. MB-related readmission was defined as hospital readmission with a primary discharge diagnosis of MB, including gastrointestinal bleeding, intracranial bleeding, and other types of major bleeding. Stroke-related readmission was defined as hospital readmission with a primary discharge diagnosis of stroke, including ischemic stroke, hemorrhagic stroke, and systemic embolism. Additionally, we conducted a sensitivity analysis in which stroke-related hospital readmissions within 1 month of discharge of initial hospitalization were descriptively evaluated when hemorrhagic strokes were not included in the stroke events.

The MB and stroke event outcomes measured in this study are similar to that measured in the DOAC vs warfarin clinical trials^6–8^ and are consistent with DOAC FDA indications. Hospital readmission length of stay (LOS) and cost of readmission were also determined and compared. All hospital cost data were inflation-adjusted to 2017 USD using the Consumer Price Index medical component.

### Statistical analyses

Mean, standard deviation (SD), and median were provided for continuous variables. Number and percentage were provided for categorical variables. Bivariate comparisons of baseline demographics, patient clinical characteristics, hospital characteristics, and readmission outcome measurements were provided, with appropriate tests (e.g. ANOVA test, chi-square test) used based on the distribution of the measure.

Propensity score matching (PSM) 1:1 was used to control for confounders, including age, gender, race, payer type, Charlson Comorbidity Index, CHA_2_DS_2_ -VASc score, HAS-BLED score, stroke/bleeding history, and hospital characteristics, including index hospitalization LOS and cost, when comparing each of the other OAC cohorts vs the apixaban cohort (reference) separately. Thus, there were the following matched cohorts: warfarin vs apixaban, rivaroxaban vs apixaban, and dabigatran vs apixaban. Specifically, in the PSM, each subject in the apixaban cohort was matched to a subject in one of the other OAC cohorts with the closest propensity score. The matched subjects were required to have propensity scores within 0.001 of each other (matching caliper). This caliper was selected to ensure that the matched study cohorts would have similar key patient characteristics. After PSM, no statistically significant differences (i.e. all *p* > .05) were observed for the key index and baseline measures between each of the other OAC cohorts and their matched apixaban cohort.

Additionally, after PSM, logistic regression analyses were carried out on the matched patient cohorts to further evaluate the potential impact of treatment with each of the OACs vs apixaban on 1-month all-cause, MB-related, and stroke-related readmissions. Only the index drugs were used as covariates, since other patient characteristics were similar after the PSM. *p*-Values were determined by analyses of Maximum Likelihood Estimates. Generalized linear model (GLM) regression analyses were also carried out on the matched patient cohorts to examine the potential impact of treatment on the average readmission LOS per patient related to 1-month all-cause, MB-related, and stroke-related readmissions (only the index drugs were used as covariates in the regressions). Two-part regression analyses were conducted to examine the differences in all-cause, MB-related, and stroke-related readmission hospital costs between each of the OAC cohorts and the apixaban cohort. In the two-part models, the first part was multivariable logistic regression, which was used to evaluate the impact of OAC treatment on the risk of all-cause, MB-related, and stroke-related readmissions. The second part was a GLM with log transformation and gamma distribution applied to the corresponding hospital cost data among readmissions. Thus, for example, for the MB-related cost evaluation, this evaluated the incremental MB-related cost among patients with MB-related readmissions. Then the odds ratio estimated from the first part was combined with the incremental MB-related costs estimated from the second part to estimate the incremental MB-related cost among all patients. Such two-part calculations were carried out in 1,000 cycles of random bootstrapping resampling to generate 1,000 such estimates. The univariate statistics of the 1,000 incremental MB-related costs among all patients were used to evaluate the MB-related cost distribution. The 2.5-percentile and 97.5-percentile of the incremental MB-related costs estimated from the 1,000 cycles of bootstrapping were used to represent the lower and upper level of the 95% confidence interval.

Sensitivity analyses were additionally conducted in which 3-month readmission rates, associated LOS, and costs of all-cause, MB-related, and stroke-related readmissions were compared between each of the other OAC cohorts and the apixaban cohort.

## Results

### Characteristics of the unmatched study cohorts

Demographics, patient clinical characteristics, and hospital characteristics of the unmatched hospitalized NVAF patients treated with the four different OACs are shown in Table 1. Of the overall study population (*n* = 529,983), 104,937 (19.8%) were treated with apixaban, 284,001 (53.6%) were treated with warfarin, 99,998 (18.9%) were treated with rivaroxaban, and 41,047 (7.7%) were treated with dabigatran during index hospitalizations. Patients who received warfarin were significantly older (75.6 years) than those who received apixaban (73.5 years), dabigatran (72.7 years), and rivaroxaban (71.4 years) (*p* < .001 across the four study cohorts). Additionally, patients treated with warfarin exhibited the greatest level of comorbidity as measured by Charlson Comorbidity Index score (warfarin: 2.7, apixaban: 2.5, rivaroxaban: 2.2, dabigatran: 2.2, *p* < .001). Patients treated with warfarin also had significantly higher risk of stroke and bleeding as measured by mean CHA_2_DS_2_-VASc score (warfarin: 4.3, apixaban: 4.0, rivaroxaban: 3.7, dabigatran: 3.9, *p* < .001) and mean HAS-BLED score (warfarin: 3.3, apixaban: 3.1, rivaroxaban: 2.9, dabigatran: 3.0, *p* < .001), respectively.

**Table 1.** Demographics, clinical characteristics, and hospital characteristics of unmatched study cohorts.

	Apixaban (*n* = 104,937)		Warfarin (*n* = 284,001)		Rivaroxaban (*n* = 99,998)		Dabigatran (*n* = 41,047)		*p*-value*
Demographics									
Age (years)									
Mean (SD)	73.5 (11.6)		75.6 (10.8)		71.4 (12.0)		72.7 (11.4)		<.001
Median	75		77		73		74		
Age group, *n* %									<.001
18–34 years	393	0.4	591	0.2	614	0.6	163	0.4	
35–44 years	1,396	1.3	2,157	0.8	1,840	1.8	469	1.1	
45–54 years	5,047	4.8	9,979	3.5	6,732	6.7	2,211	5.4	
55–64 years	15,111	14.4	31,942	11.3	17,464	17.5	6,448	15.7	
65–74 years	28,972	27.6	69,924	24.6	29,122	29.1	12,093	29.5	
≥75 years	54,018	51.5	169,408	59.7	44,226	44.2	19,663	47.9	
Gender, *n* %									<.001
Female	51,102	48.7	129,848	45.7	45,713	45.7	17,931	43.7	
Male	53,835	51.3	154,153	54.3	54,285	54.3	23,116	56.3	
Race, *n* %									<.001
White	87,786	83.7	230,525	81.2	81,718	81.7	33,769	82.3	
Black	7,513	7.2	20,323	7.2	7,087	7.1	2,594	6.3	
Other	9,638	9.2	33,153	11.7	11,193	11.2	4,684	11.4	
Payer type, *n* %									<.001
Commercial	16,486	15.7	26,586	9.4	19,384	19.4	7,131	17.4	
Medicare	80,684	76.9	235,609	83.0	72,127	72.1	31,089	75.7	
Medicaid	4,166	4.0	11,555	4.1	4,919	4.9	1,463	3.6	
Others	3,601	3.4	10,251	3.6	3,568	3.6	1,364	3.3	
Clinical characteristics									
Charlson Comorbidity Index (CCI)									
								
								
Mean (SD)	2.5 (2.1)		2.7 (2.1)		2.2 (2.0)		2.2 (2.0)		<.001
Median	2		2		2		2		
CHADS_2_ score									
Mean (SD)	2.5 (1.3)		2.8 (1.3)		2.3 (1.3)		2.5 (1.3)		<.001
Median	2		3		2		2		
CHA_2_DS_2_ -VASc score									
Mean (SD)	4.0 (1.7)		4.3 (1.6)		3.7 (1.8)		3.9 (1.7)		<.001
Median	4		4		4		4		
HAS-BLED score									
Mean (SD)	3.1 (1.2)		3.3 (1.2)		2.9 (1.2)		3.0 (1.2)		<.001
Median	3		3		3		3		
Stroke/bleeding history, *n* %									
Prior stroke	2,448	2.3	7953	2.8	2,259	2.3	1,101	2.7	<.001
Stroke in index hospitalization	7,918	7.6	20,562	7.2	5,753	5.8	2,259	5.5	<.001
Prior bleeding	1,962	1.9	7,951	2.8	2,052	2.1	820	2.0	<.001
Bleeding in index hospitalization	9,025	8.6	37,110	13.1	9,022	9.0	3,187	7.8	<.001
Hospital characteristics									
Location, *n* %									<.001
Urban	90,867	86.6	247,379	87.1	87,540	87.5	35,758	87.1	
Rural	14,070	13.4	36,622	12.9	12,458	12.5	5,289	12.9	
Teaching status, *n* %									<.001
Yes	41,751	39.8	112,480	39.6	38,391	38.4	16,753	40.8	
No	63,186	60.2	171,521	60.4	61,607	61.6	24,294	59.2	
Bed size, *n* %									<.001
0–99 beds	6,092	5.8	17,252	6.1	5,756	5.8	2,429	5.9	
100–199 beds	16,177	15.4	42,431	14.9	14,679	14.7	5,807	14.2	
200–299 beds	19,867	18.9	52,296	18.4	19,252	19.3	7,971	19.4	
300–399 beds	18,699	17.8	50,241	17.7	17,947	18.0	7,306	17.8	
400–499 beds	13,832	13.2	38,885	13.7	12,030	12.0	5,281	12.9	
≥500 beds	30,270	28.9	82,896	29.2	30,334	30.3	12,253	29.9	

*p-values are for comparison across the four study populations treated with the different direct oral anticoagulants.

Abbreviations. CHADS_2_, congestive heart failure, hypertension, age = 75 years, diabetes mellitus, stroke; CHA_2_DS_2_-VASc, congestive heart failure, hypertension, age = 75 years, diabetes mellitus, stroke, vascular disease; HAS-BLED, hypertension, abnormal renal/liver function, stroke, bleeding history or predisposition; SD, standard deviation.

### Matched study cohorts: warfarin vs apixaban

#### Characteristics of study cohorts

Demographics, patient clinical characteristics, and hospital characteristics of the matched warfarin (*n* = 69,765) and apixaban (*n* = 69,765) treated cohorts are shown in Table 2. After PSM, the patient demographics, clinical characteristics, and hospital characteristics of the two study cohorts were similar and not statistically significantly different (all *p* > .05). The mean age of both study cohorts was 76.0 years, approximately half were female, 84.4–84.6% were white, and the majority (84%) had Medicare coverage. Greater than 85% of either study cohort received care in urban hospitals, and the majority of hospitals were large in size (>300 beds). The mean LOS for index hospitalizations was 4.4 days for both study cohorts and the average index hospital cost was $9,177 for warfarin-treated patients and $9,220 for apixaban-treated patients.

**Table 2.** Demographics, clinical characteristics, and hospital characteristics of matched study cohorts.

	Warfarin (*n* = 69,765)		Apixaban (*n* = 69,765)		*p*-value	Rivaroxaban (*n* = 59,747)		Apixaban (*n* = 59,747)		*p*-value	Dabigatran (*n* = 39,604)		Apixaban (*n* = 39,604)		*p*-value
Demographics															
Age (years)															
Mean (SD)	76.0 (9.8)		76.0 (9.8)		.64	72.7 (11.2)		72.8 (11.5)		.09	72.9 (11.3)		72.9 (11.8)		.72
Median	77		77			74		74			74		74		
Gender, *n* %					.42					.81					.52
Female	35,391	50.7	35,239	50.5		28,709	48.1	28,750	48.1		17,626	44.5	17,717	44.7	
Male	34,374	49.3	34,526	49.5		31,038	52.0	30,997	51.9		21,978	55.5	21,887	55.3	
Race, *n* %					.36					.12					.96
White	59,045	84.6	58,851	84.4		50,383	84.3	50,625	84.7		32,869	83.0	32,861	83.0	
Black	4,570	6.6	4,647	6.7		4,271	7.2	4,117	6.9		2,534	6.4	2,552	6.4	
Other	6,150	8.8	6,267	9.0		5,093	8.5	5,005	8.4		4,201	10.6	4,191	10.6	
Payer type, *n* %					.19					.95					.95
Commercial	7,074	10.1	7,034	10.1		10,178	17.0	10,107	16.9		6,759	17.1	6,752	17.1	
Medicare	58,582	84.0	58,467	83.8		45,080	75.5	45,167	75.6		30,122	76.1	30,108	76.0	
Medicaid	2,073	3.0	2,090	3.0		2,385	4.0	2,372	4.0		1,406	3.6	1,436	3.6	
Others	2,036	2.9	2,174	3.1		2,104	3.5	2,101	3.5		1,317	3.3	1,308	3.3	
Clinical characteristics															
Charlson Comorbidity Index (CCI)															
Mean (SD)	2.3 (1.7)		2.3 (1.7)		.45	2.1 (1.9)		2.1 (1.9)		.62	2.2 (2.0)		2.2 (2.0)		.85
Median	2		2			2		2			2		2		
CHADS_2_ score															
Mean (SD)	2.6 (1.2)		2.6 (1.2)		.31	2.4 (1.2)		2.3 (1.2)		.12	2.5 (1.3)		2.5 (1.3)		.26
Median	3		3			2		2			2		2		
CHA_2_DS_2_ -VASc score															
Mean (SD)	4.2 (1.6)		4.2 (1.6)		.84	3.8 (1.6)		3.8 (1.6)		.42	3.9 (1.7)		3.9 (1.7)		.51
Median	4		4			4		4			4		4		
HAS-BLED score															
Mean (SD)	3.1 (1.1)		3.1 (1.1)		1.00	2.9 (1.1)		2.9 (1.1)		1.00	3.0 (1.2)		3.0 (1.2)		.18
Median	3		3			3		3			3		3		
Stroke/bleeding history, *n* %															
Prior stroke	1,369	2.0	1,444	2.1	.15	208	.4	208	.4	1.00	998	2.5	998	2.5	1.00
Stroke in index hospitalization	4,855	7.0	4,845	6.9	.92	3,401	5.7	3,300	5.5	.20	2,237	5.7	2,307	5.8	.28
Prior bleeding	1,270	1.8	1,206	1.7	.19	755	1.3	766	1.3	.78	756	1.9	739	1.9	.66
Bleeding in index hospitalization	5,187	7.4	5,201	7.5	.89	3,762	6.3	3,666	6.1	.25	3,087	7.8	3,099	7.8	.87
Hospital characteristics															
Location, *n* %					.31					.36					.55
Urban	59,770	85.7	59,903	85.9		51,724	86.6	51,831	86.8		34,410	86.9	34,353	86.7	
Rural	9,995	14.3	9,862	14.1		8,023	13.4	7,916	13.3		5,194	13.1	5,251	13.3	
Teaching status, *n* %					.09					.23					.58
Yes	26,469	37.9	26,778	38.4		22,676	38.0	22,476	37.6		15,984	40.4	15,908	40.2	
No	43,296	62.1	42,987	61.6		37,071	62.1	37,271	62.4		23,620	59.6	23,696	59.8	
Bed size, *n* %					.10					.44					.94
0–99 beds	4,524	6.5	4,408	6.3		3,670	6.1	3,648	6.1		2,378	6.0	2,432	6.1	
100–199 beds	11,194	16.1	11,308	16.2		9,372	15.7	9,414	15.8		5,699	14.4	5,733	14.5	
200–299 beds	13,468	19.3	13,202	18.9		11,611	19.4	11,618	19.5		7,627	19.3	7,551	19.1	
300–399 beds	12,630	18.1	12,544	18.0		10,810	18.1	10,984	18.4		7,085	17.9	7,110	18.0	
400–499 beds	9,396	13.5	9,354	13.4		7,506	12.6	7,286	12.2		5,112	12.9	5,118	12.9	
≥500 beds	18,553	26.6	18,949	27.2		16,778	28.1	16,797	28.1		11,703	29.6	11,660	29.4	
Index hospital length of stay															
Mean (SD)	4.4 (3.3)		4.4 (3.4)		.34	4.0 (3.0)		4.0 (3.0)		.56	4.5 (4.0)		4.5 (4.1)		.63
Median	4		3			3		3			3		3		
Index hospital cost															
Mean (SD)	$9,177 ($7,350)		$9,220 ($7,398)		.28	$8,569 ($6,704)		$8,580 ($6,714)		.78	$11,009 ($12,038)		$11,044 ($13,145)		.69
Median	$6,918		$7,032			$6,558		$6,591			$7,148		$7,194		

Abbreviations. CHADS_2_, congestive heart failure, hypertension, age = 75 years, diabetes mellitus, stroke; CHA_2_DS_2_-VASc, congestive heart failure, hypertension, age = 75 years, diabetes mellitus, stroke, vascular disease; HAS-BLED, hypertension, abnormal renal/liver function, stroke, bleeding history or predisposition; SD, standard deviation.

#### Unadjusted readmission outcomes after PSM

After PSM, the proportions of patients with all-cause (14.7% vs 14.1%, *p* < .001), MB-related (1.2% vs 0.9%, *p* < .001), and stroke-related (0.42% vs 0.32%, *p* = .001) readmissions within 1 month of index hospitalization were all significantly greater among the warfarin cohort than the apixaban cohort (Table 3). Also, the average LOS among all readmission categories was significantly longer and the associated average hospital costs were significantly higher for patients treated with warfarin compared to patients treated with apixaban (Table 3). These results were across all patients in study cohorts, including patients where the LOS and hospital cost of patients without readmissions were equal to 0 days and $0, respectively.

**Table 3.** Unadjusted rates of readmissions, associated LOS, and hospital costs per patient of matched study cohorts.

Readmission category	Warfarin (*n* = 69,765)		Apixaban (*n* = 69,765)		*p-*value	Rivaroxaban (*n* = 59,747)		Apixaban (*n* = 59,747)		*p*-value	Dabigatran (*n* = 39,604)		Apixaban (*n* = 39,604)		*p*-value
All-cause, *n* %	10,280	14.7	9,844	14.1	<.001	8,155	13.7	7,778	13.0	.001	5,196	13.1	5,353	13.5	.101
All-cause LOS (days)															
Mean	1.13		1.02		<.001	.95		.90		.004	.98		.98		.937
SD	3.93		3.62			3.50		3.38			3.64		3.60		
All-cause hospital cost															
Mean	$2,378		$2,251		.028	$2,053		$2,003		.322	$2,178		$2,268		.194
SD	$10,180		$9,865			$8,287		$8,943			$8,972		$10,347		
MB-related, *n* %	834	1.2	651	.9	<.001	730	1.2	452	.8	<.001	392	1.0	348	.9	.104
MB-related LOS (days)															
Mean	.07		.06		<.001	.07		.04		<.001	.06		.05		.111
SD	.86		.79			.75		.70			.84		.80		
MB-related hospital cost															
Mean	$157		$133		.042	$147		$105		<.001	$160		$135		.235
SD	$1,948		$2,511			$1,816		$2,311			$3,060		$2,820		
Stroke-related, *n* %	294	.4	222	.3	.001	158	.3	153	.3	.776	116	.3	103	.3	.379
Stroke-related LOS (days)															
Mean	.03		.02		.003	.02		.01		.466	.02		.02		.158
SD	.56		.43			.42		.38			.77		.43		
Stroke-related hospital cost															
Mean	$63		$45		.031	$37		$38		.925	$51		$40		.301
SD	$1,851		$1,304			$1,213		$1,285			$1,598		$1,383		

These results were across all patients in each of the paired study cohorts, including patients where the LOS and hospital cost of patients without readmissions were equal to 0 days and $0, respectively. Some patients may have had both MB and stroke events (not mutually exclusive).

Abbreviations. LOS, length of stay; MB, major bleeding; SD, standard deviation.

In the sensitivity analysis, in which hemorrhagic strokes were not included in the stroke events, the direction of the findings was relatively consistent with the findings from the default analysis, with the warfarin cohort having a greater proportion of patients with stroke-related readmissions within 1 month of index hospitalization than the apixaban cohort (0.36% vs 0.29%, *p* = .013).

#### Regression adjusted readmission outcomes after PSM

**Figure 1.**
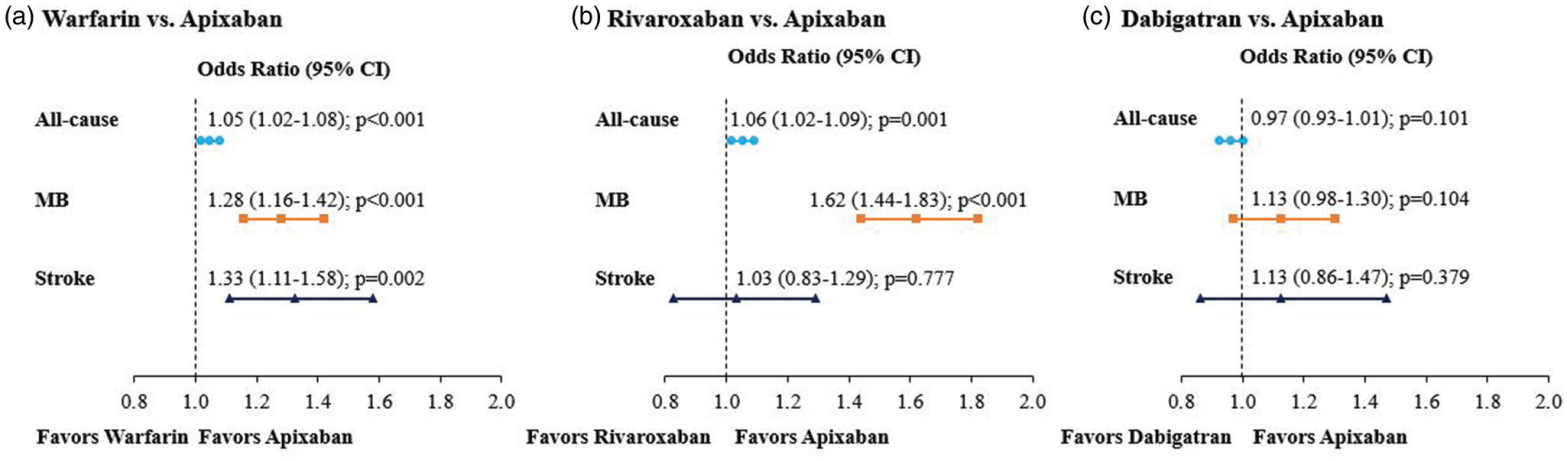
Regression adjusted risks of all-cause, MB-related, and stroke-related readmissions for matched study cohorts. CI, confidence interval; MB, major bleeding.

The regression analyses showed that NVAF patients treated with warfarin vs apixaban had significantly greater risk of all-cause readmission (OR = 1.05; 95% CI = 1.02–1.08; *p* < .001), MB-related readmission (OR = 1.28; 95% CI = 1.16–1.42; *p* < .001), and stroke-related readmission (OR = 1.33; 95% CI = 1.11–1.58; *p* = .002) within 1 month of index hospitalization (Figure 1a). For all-cause readmission, warfarin-treated patients had on average a 0.11 day longer LOS per patient (*p* < .001) and a hospital cost that was on average $134 higher per patient (*p* = .010) than apixaban-treated patients (Table 4). For MB-related readmissions, warfarin- vs apixaban-treated patients had on average a 0.017 day longer LOS per patient (*p* < .001) and a hospital cost that was on average $25 higher per patient (*p* = .036) (Table 4). For stroke-related readmissions, warfarin- vs apixaban-treated patients had on average a 0.008 day longer LOS per patient (*p* = .003) and a hospital cost that was on average $19 higher per patient (*p* = .018) (Table 4). These results were across all patients in the study cohorts, including patients where the LOS and hospital cost of patients without readmissions were equal to 0 days and $0, respectively.

**Table 4.** Regression analyses─LOS and associated hospital cost for readmissions per patient of matched study cohorts: other oral anticoagulants (OACs) vs apixaban.

Readmission category	Warfarin vs Apixaban	*p*-value	Rivaroxaban vs Apixaban	*p*-value	Dabigatran vs Apixaban	*p-*value
All-cause LOS (days)						
Other OAC	1.13		.95		.978	
Apixaban	1.02		.90		.976	
Mean difference	.11	<.001	.06	.004	.002	.937
95% CI	.07–.15		.02–.10		−.048–.053	
All-cause hospital cost						
Other OAC	$2,387		$2,058		$2,194	
Apixaban	$2,253		$2,003		$2,270	
Mean difference	$134	.010	$55	.280	−$76	.246
95% CI	$32–$243		−$44–$156		−$211–$52	
MB-related LOS (days)						
Other OAC	.073		.065		.064	
Apixaban	.056		.044		.055	
Mean difference	.017	<.001	.021	<.001	.009	.111
95% CI	.008–.026		.013–.029		−.002–.021	
MB-related hospital cost						
Other OAC	$158		$148		$162	
Apixaban	$132		$105		$135	
Mean difference	$25	.036	$43	<.001	$27	.214
95% CI	$2–$50		$16–$69		−$15–$71	
Stroke-related LOS (days)						
Other OAC	.027		.016		.022	
Apixaban	.019		.014		.016	
Mean difference	.008	.003	.002	.466	.006	.158
95% CI	.003–.013		−.003–.006		−.002–.015	
Stroke-related hospital cost						
Other OAC	$64		$38		$51	
Apixaban	$45		$38		$40	
Mean difference	$19	.018	$0	.956	$11	.294
95% CI	$3–$38		−$14–$15		−$9–$35	

These results were across all patients in each of the paired study cohorts, including patients where the LOS and hospital cost of patients without readmissions were equal to 0 days and $0, respectively.

Abbreviations. LOS, length of stay; MB, major bleeding.

### Matched study cohorts: rivaroxaban vs apixaban

#### Characteristics of study cohorts

Demographics, patient clinical characteristics, and hospital characteristics of the matched rivaroxaban (*n* = 59,747) and apixaban (*n* = 59,747) treated cohorts are shown in Table 2. After PSM, the patient demographics, clinical characteristics, and hospital characteristics of the two study cohorts were similar and not statistically significantly different (all *p* > .05). The mean age of the rivaroxaban cohort was 72.7 years and 72.8 years for the apixaban cohort; approximately half were female in both cohorts, 84.3–84.7% were white, and the majority (∼75%) had Medicare coverage. Greater than 86% of either study cohort received care in urban hospitals, and the majority of hospitals were large in size (>300 beds). The mean LOS for index hospitalizations was 4.0 days for both study cohorts and the average index hospital cost was $8,569 for rivaroxaban-treated patients and $8,580 for apixaban-treated patients.

#### Unadjusted readmission outcomes after PSM

After PSM, the proportions of patients with all-cause (13.7% vs 13.0%, *p* = .001) and MB-related (1.2% vs 0.8%, *p* < .001) readmissions within 1 month of index hospitalization were significantly greater among the rivaroxaban cohort than the apixaban cohort; the rates of stroke-related readmissions did not significantly differ between cohorts (0.3% vs 0.3%, *p* = .776) (Table 3). The mean LOS for all-cause readmissions was significantly longer and the associated hospital costs were numerically but not significantly higher for patients treated with rivaroxaban compared to patients treated with apixaban (Table 3). The mean LOS for MB-related readmissions was significantly longer and the associated hospital cost significantly higher for patients treated with rivaroxaban compared to patients treated with apixaban (Table 3). The mean LOS and associated hospital cost for stroke-related readmissions did not significantly differ between cohorts (Table 3). These results were across all patients in study cohorts, including patients where the LOS and hospital cost of patients without readmissions were equal to 0 days and $0, respectively.

In the sensitivity analysis, in which hemorrhagic strokes were not included in the stroke events, the direction of the findings was relatively consistent with the findings from the default analysis, with no significant differences in the rates of stroke-related readmissions between the rivaroxaban and apixaban cohorts (0.23% vs 0.23%, *p* = .952).

#### Regression adjusted readmission outcomes after PSM

The regression analyses showed that NVAF patients treated with rivaroxaban vs apixaban had significantly greater risks of all-cause readmission (OR = 1.06; 95% CI = 1.02–1.09; *p* = .001) and MB-related readmission (OR = 1.62; 95% CI = 1.44–1.83; *p* < .001); the risks of stroke-related readmission were similar for study cohorts (OR = 1.03; 95% CI = 0.83–1.29; *p* = .777) (Figure 1b). For all-cause readmissions, rivaroxaban-treated patients had on average a 0.06 day longer LOS per patient (*p* = .004) and a hospital cost that was numerically higher by $55 per patient (*p* = .280) than apixaban-treated patients, but this difference was not statistically significant (Table 4). For MB-related readmissions, rivaroxaban- vs apixaban-treated patients had on average a 0.021 day longer LOS per patient (*p* < .001) and a hospital cost that was on average $43 higher per patient (*p* < .001) (Table 4). For stroke-related readmissions, rivaroxaban- vs apixaban-treated patients had similar LOSs and hospital costs (Table 4). These results were across all patients in study cohorts, including patients where the LOS and hospital cost of patients without readmissions were equal to 0 days and $0, respectively.

### Matched study cohorts: dabigatran vs apixaban

#### Characteristics of study cohorts

Demographics, patient clinical characteristics, and hospital characteristics of the matched dabigatran (*n* = 39,604) and apixaban (*n* = 39,604) treated cohorts are shown in Table 2. After PSM, the patient demographics, clinical characteristics, and hospital characteristics of the two study cohorts were similar and not statistically significantly different (all *p* > .05). For both study cohorts, the mean age was 72.9 years, approximately 45% were female, 83% were white, and the majority (76%) had Medicare coverage. Greater than 86% of either study cohort received care in urban hospitals and the majority of hospitals were large in size (>300 beds). The mean LOS for index hospitalizations was 4.5 days for both study cohorts and the average index hospital cost was $11,009 for dabigatran-treated patients and $11,044 for apixaban-treated patients.

#### Unadjusted readmission outcomes after PSM

After PSM, the proportions of patients with all-cause (13.1% vs 13.5%, *p* = .101), MB-related (1.0% vs 0.9%, *p* = .104), and stroke-related (0.3% vs 0.3%, *p* = .379) readmissions within 1 month of index hospitalization were similar among the dabigatran cohort and the apixaban cohort (Table 3). The mean LOSs and associated hospital costs for readmissions also did not significantly differ between cohorts (Table 3).

In the sensitivity analysis, in which hemorrhagic strokes were not included in the stroke events, the direction of the findings was relatively consistent with the findings from the default analysis, with no significant differences in the rates of stroke-related readmissions between the dabigatran and apixaban cohorts (0.26% vs 0.22%, *p* = .280).

#### Regression adjusted readmission outcomes after PSM

The regression analyses showed similar results as the unadjusted data for the dabigatran and apixaban cohorts with no significant differences in the measured outcomes (Figure 1c, Table 4).

#### Sensitivity analyses

The findings of the sensitivity analyses in which the study outcomes were evaluated after 3-months were generally consistent with the results of the default analysis.

## Discussion

In this analysis of nearly 530,000 patients with NVAF who were hospitalized from January 2013 to June 2017 and treated with OACs, just over one-half were treated with warfarin (53.6%), 19.8% were treated with apixaban, 18.9% with rivaroxaban, and 7.7% with dabigatran. While the Premier Hospital database is nationally representative, these findings may not reflect the national prescriptions rates of OACs, especially in the outpatient setting, as well as in the most recent years in light of the rapidly changing OAC prescription patterns in the US. Similar to that observed in prior studies conducted in the real-world setting, NVAF patients treated with warfarin were generally older, had greater comorbidities, and had higher stroke and bleeding risks than patients treated with DOACs^10^^,^^12^. Although the differences were relatively small regarding mean ages and general comorbidity among the DOAC cohorts in this study, apixaban was more frequently observed as a treatment for NVAF patients who were older and sicker, which has also been seen in prior studies^10^^,^^12^^,^^20^. These observations show that, despite the greater efficacy and safety of some DOACs, warfarin remains the most common OAC prescribed to NVAF patients; meanwhile, the usage of DOACs, in particular apixaban and rivaroxaban, appears to be increasing in the US NVAF population.

Among the NVAF study cohorts treated with OACs, the unadjusted rates of 1-month readmissions for any cause for patients matched with similar characteristics ranged between 13.0% and 14.7%. These rates were generally consistent with our previous study, in which the overall all-cause hospital readmission rate was ∼ 15% among NVAF patients identified from the Premier Hospital database between January 2012 and March 2014^20^. Our all-cause readmission rates are also similar to the findings of two nationwide studies of AF patients^21^^,^^22^. In the first, Munir et al.^21^ reported that among 388,340 hospitalized AF patients (≥18 years of age) identified from the Nationwide Readmissions Database (2013), 15.1% were readmitted to the hospital for any cause within 30 days. The second study was of Medicare beneficiaries (≥65 years of age) hospitalized for AF by Freeman et al.^22^, and a 1-month all-cause readmission rate of 13.9% was reported for 2013; the rates ranged between 15.3% and 15.6% in the others years analyzed (1999–2012).

In our study, the unadjusted rates of 1-month MB-related readmissions ranged between 0.8% and 1.2%; rates of stroke-related readmissions were 0.3% for all DOAC cohorts and 0.4% for the warfarin cohort. The findings of the regression analyses showed that, compared to NVAF patients treated with apixaban, those treated with warfarin had a 5% significantly increased risk for all-cause readmission, a 28% significantly increased risk for MB-related readmission, and a 33% increased risk for stroke-related readmission. NVAF patients treated with rivaroxaban vs apixaban had a 6% significantly increased risk for all-cause readmission and a 62% increased risk for MB-related readmission, which in regard to the latter is directionally consistent with the finding of our previous study^20^. Also consistent with previous findings, we did not find significant differences in readmission outcomes for NVAF patients treated with dabigatran vs apixaban^20^. For this study we specifically were concerned with hospital readmission outcomes, but our findings between the warfarin and apixaban cohorts and the rivaroxaban and apixaban cohorts provide further evidence that apixaban treatment may be associated with better clinical outcomes than these other OACs. These findings are aligned with those of several real-world evidence studies^9–19^, as well as network meta-analyses of clinical trial data^23^^,^^24^, in which all have generally found apixaban to have the better safety profile (i.e. lowest bleeding risk) compared to other OACs.

Regarding hospital LOS and costs for MB-related readmissions, compared to those treated with apixaban, patients treated with either warfarin or rivaroxaban who had similar patient/hospital characteristics had significantly longer LOSs and higher costs. Warfarin-treated patients also had significantly longer LOSs and higher costs for all-cause and stroke-related readmissions compared to patients treated with apixaban; rivaroxaban-treated patients had a significantly longer LOS associated with all-cause readmissions as well. In this study we did not break down hospital costs into categories, such as pharmacy costs, nor did we evaluate specific costs of the OACs. In the hospital setting, the price of OACs may be subject to many variations, including regional differences and hospital contracting with manufacturers, etc.; however, since in the inpatient setting patients received OACs for only a few days, such OAC drug costs likely represented only a very small portion of the overall hospital cost.

In the analyses of readmission LOSs and costs, the comparisons were carried out among all patients in each of the paired study cohorts, where the LOS and hospital cost of patients without readmissions were equal to 0 days and $0, respectively. Thus, these data are quite relevant on the population level of NVAF patients since they display the incremental hospital resource use and cost burden across all NVAF patients treated with each of the other OACs vs apixaban. For example, the difference in LOS for MB-related readmissions between the apixaban and warfarin cohorts (total patient count = 139,530) was an average of 0.017 days and the cost difference was an average of $25 per patient. The differences may appear to be numerically small; however, considering that there may be many thousands of such patients treated with warfarin in the real-world settings, the all-cause readmission cost differences can be substantial (e.g. for the 69,765 matched warfarin treated patients, the all-cause readmission cost difference vs apixaban treated patients amounts to $9,348,510 [69,765 × $134]). Such data may be helpful for providers and payers when evaluating the potential impact of particular treatment options on their entire NVAF populations that they provide care. Moreover, the results of this study may be of particular importance; based on data between 1 January 2013 and 31 November 2013 captured in the Nationwide Readmission Database, of 12,533,551 all-cause hospitalizations 14.5% were associated with readmission within 30 days, at a total cost of nearly 51 billion USD (Medicare 29.6 billion; non-Medicare: 21 billion)^25^.

The nationwide study of Freeman et al.^22^ of Medicare insured AF patients reported that between 1999 and 2013 the median LOS for readmissions (any cause) remained consistent at 3 days; however, there was a 60% increase in per event median readmission costs, from $2,932 in 1999 to $4,719 in 2013. Further study of this cost trend of readmissions for AF patients is needed in the more recent years of Medicare inpatient cost containment policies. The per event readmission costs for MB and stroke among NVAF patients have not been well documented in the published literature. However, a database claims study of patients with an AF diagnosis and a hospitalization for an MB event reported a mean hospital cost of $28,509 per patient (2014 USD)^26^. Costs for hospital readmissions of NVAF patients are substantial and possibly increasing. Alongside the growing prevalence of NVAF, it is worthwhile to identify those treatments that have the potential to minimize this economic burden, especially related to MB.

## Limitations

This study was a retrospective, observational, matched cohort analysis that used a nationally representative hospital database with limitations that should be considered when interpreting the results. The data on patient records in the Premier hospital database are the costs to hospitals only, therefore outpatient healthcare utilization and costs not received in a hospital are excluded. Thus, in the case of warfarin, the outpatient routine monitoring care and associated costs were not captured in our analysis. Additionally, time-in-therapeutic range data were not available in the data source and thus were not evaluated for patients treated with warfarin. While the Premier Hospital database contains information from a substantial number of hospitals across the US, the database populations may not be representative of the entire US population of NVAF patients. Also, only readmissions to the same hospital or hospital system within the Premier network could be identified in the database, which may have led to an underestimation of actual readmission rates. MB-related and stroke-related readmissions were evaluated by the primary hospital discharge diagnoses for the readmissions, which may or may not fully capture the entire cause of readmissions. More specific types of stroke, such as lacunar and large vessel embolism, were not available in the data source and were not evaluated in the study. As with all studies relying on administrative billing information, there may have been inaccuracies from hospital, billing, and coding errors, as well as missing data. Although PSM was used to control for multiple confounders, there is potential for residual bias in this study. For instance, the PSM did not take into account other confounding factors that were not included in the covariate list of the PSM process. Lastly, no causal relationship between OAC treatment and outcomes can be concluded based on this retrospective observational analysis.

## Conclusions

According to this large-scale, retrospective, real-world, hospital analysis of NVAF patients, after controlling for differences in patient and hospital characteristics, apixaban treatment was associated with significantly lower all-cause, MB-related, and stroke-related hospital readmission risk than warfarin and significantly lower all-cause and MB-related hospital readmission risk, but not stroke-related readmission, than rivaroxaban. Significant differences in the risks of all-cause, MB-related, and stroke-related readmissions were not observed between the apixaban and dabigatran cohorts. Apixaban was also associated with significantly lower costs for all-cause readmission (vs warfarin only), MB-related readmission (vs warfarin and rivaroxaban), and stroke-related readmission (vs warfarin only). The results of this study may be helpful to guide hospitals, payers, patients, and other stakeholders in determining the optimal oral anticoagulation therapy that provides the most benefit to NVAF patients, while reducing the hospital resource and economic burden.

## Transparency

### Declaration of financial/other relationships

SD is a consultant for Pfizer, Bristol-Myers Squibb, and Portola Pharmaceuticals. CLB, JM, OD, and CR are employees and stockholders of Pfizer. ADD, LR, and TP are employees of Bristol-Myers Squibb. MLS and JL are employees of Novosys Health, which has received research funds from Pfizer and Bristol-Myers Squibb in connection with conducting this study and development of this manuscript. Peer reviewers on this manuscript have no relevant financial or other relationships to disclose.
